# Association of Educational Attainment and Race/Ethnicity With Exposure to Tobacco Advertisement Among US Young Adults

**DOI:** 10.1001/jamanetworkopen.2019.19393

**Published:** 2020-01-17

**Authors:** Shervin Assari

**Affiliations:** 1 Department of Family Medicine, Charles R. Drew University of Medicine and Science, Los Angeles, California

## Abstract

**Importance:**

Associations of educational attainment with improved health outcomes have been found to be weaker among racial/ethnic minority groups compared with those among the racial/ethnic majority group. Recent research has also documented higher than expected prevalence of smoking in highly educated African American and Hispanic adults.

**Objective:**

To compare the association of educational attainment with exposure to tobacco advertisements among racial/ethnic groups of US young adults.

**Design, Setting, and Participants:**

This cross-sectional study included data from 6700 young adults who participated in wave 1 of the Population Assessment of Tobacco and Health Study, a nationally representative survey of US adults in 2013. Educational attainment was classified as less than high school diploma, high school graduate, or college graduate. Analysis was conducted between September 20 and October 4, 2019.

**Main Outcomes and Measures:**

The independent variable was educational attainment (less than high school diploma, high school graduate, and college graduate). The dependent variable was any exposure to tobacco advertisements in the past 12 months. Race/ethnicity, age, sex, poverty status, unemployment, and region were the covariates. Binary logistic and Poisson regression were used to analyze the data.

**Results:**

The study included 6700 participants (3366 [50.2%] men) between ages 18 and 24 years. Most participants were non-Hispanic (5257 participants [78.9%]) and white (5394 participants [80.5%]), while 1443 participants (21.5%) were Hispanic. Educational levels included 1167 participants (17.4%) with less than a high school diploma, 4812 participants (71.8%) who were high school graduates, and 4812 participants (10.8%) who were college graduates. A total of 4728 participants (70.6%) reported exposure to tobacco advertisements in the past 12 months. Exposure to tobacco advertising was reported by 383 participants (53.1%) who were college graduates, 3453 participants (71.8%) who were high school graduates, and 892 participants (76.4%) with less than high school educational attainment. In regression analysis, high school graduation (odds ratio, 0.79; 95% CI, 0.68-0.92) and college graduation (odds ratio, 0.46; 95% CI, 0.39-0.54) were associated with lower odds of exposure to tobacco advertisements compared with young adults with lower educational attainment. Compared with non-Hispanic participants, high school education had a weaker protective association for tobacco advertisement exposure among Hispanic participants (odds ratio, 1.44; 95% CI, 1.03-2.01; *P* = .03), suggesting that the association of high school graduation with lower exposure to tobacco advertisement is weaker among Hispanic young adults than non-Hispanic young adults.

**Conclusions and Relevance:**

This study found that high school graduation had a weaker inverse association with tobacco advertisement exposure among Hispanic than non-Hispanic young adults. Future research should explore the role of targeted marketing strategies of the tobacco industry that largely advertise tobacco in areas with high concentrations of racial/ethnic minority groups. Future research should also evaluate the efficacy of more restrictive marketing policies on racial/ethnic disparities in tobacco use.

## Key Points

**Question:**

What are the associations of educational attainment with exposure to tobacco advertisements among racial/ethnic groups of young adults?

**Findings:**

This cross-sectional study among 6700 US young adults found a weaker inverse association of educational attainment with exposure to tobacco advertisement among Hispanic young adults compared with non-Hispanic young adults.

**Meaning:**

This finding suggests that elimination of racial/ethnic disparities in tobacco use may require more than equalizing educational attainment across racial/ethnic groups.

## Teaser

This cross-sectional study examines the association of educational attainment with exposure to tobacco advertisements among diverse racial/ethnic groups of US young adults.

## Introduction

In the United States, considerable racial/ethnic disparities exist in the burden of tobacco use.^1,2,3,4,5^ Despite some racial/ethnic minority groups, such as Hispanic and African American people, having a lower prevalence of tobacco use compared with non-Hispanic white people, racial/ethnic minority groups continue to have higher rates of adverse tobacco outcomes—a paradox well known to tobacco researchers.^3,6,7^ Owing to low access to tobacco cessation programs^3,6,7^ combined with low acceptability and trust in the health care system overall and tobacco cessation services in particular, African American and Hispanic young adults remain at an increased risk of adverse tobacco-related outcomes, such a cancer, respiratory conditions, and heart disease.^8^

Traditionally, some of the racial/ethnic differences in tobacco use have been associated with lower socioeconomic status of racial/ethnic minority groups.^9,10,11,12^ The role of socioeconomic status as a social determinant of tobacco disparities has recently increased in the United States, which can be seen as a challenge to the success of US policies in reducing tobacco use prevalence.^12,13,14^ From 1966 to 2015, cigarette smoking declined by 83% among people in the United States with college degrees. The same decline was less than half as strong (40%) among individuals who did not have a high school diploma.^15^ If the socioeconomic gap was solely responsible for racial/ethnic disparities in tobacco use, then policies aimed at the elimination of economic inequalities would have had some success in narrowing the tobacco gap across racial/ethnic and socioeconomic groups, which is clearly not the case.^12,13,14^ Thus, socioeconomic status does not seem to be the sole mediator of the racial/ethnic gap in tobacco burden. Therefore, to eliminate racial/ethnic and socioeconomic gaps, noneconomic interventions, such as restricting tobacco marketing, may be required.

Studies from 2018^16^ and 2019^17^ have shown that not all of the racial/ethnic differences in tobacco use are associated with socioeconomic status inequalities across such groups. The inverse association of educational attainment with substance use, particularly tobacco use, has been found to be smaller among racial/ethnic minority groups than among non-Hispanic white individuals.^16,17^ This is a phenomenon that I have labeled “minorities’ diminished returns.”^18,19,20^ This phenomenon refers to a weaker than expected association of educational attainment with health outcomes among racial/ethnic minority individuals, particularly Hispanic and African American individuals, compared with non-Hispanic white individuals.^18,20^ Similarly, prevalence of tobacco use would be higher than expected in highly educated Hispanic and African American individuals.^19,21,22^ Such greater-than-expected tobacco use imposes a considerable potential threat to middle-class Hispanic and African American individuals.^16,17^ Although similar patterns have been repeatedly shown for other outcomes in Hispanic^17,22^ and African American^21,23,24^ individuals, the exact mechanisms of a weaker association of educational attainment with tobacco use remain unknown, to my knowledge.

Despite replicability of studies showing diminished health outcomes associated with educational attainment among racial/ethnic minority groups,^16,17^ few studies have explored specific mechanisms for this phenomenon in the field of tobacco use.^25^ In a 2019 study,^26^ secondhand workplace exposure to cigarette smoke was higher in highly educated Hispanic and African American individuals, which was attributed to labor market discrimination, which often results in Hispanic and African American individuals working in occupations with higher stress and lower pay than non-Hispanic white individuals. Another 2019 study^25^ suggested that home smoke-free tobacco policies were associated with African American households at a lower rate than expected compared with white households. One mechanism to be examined in this study is the differential role of higher educational attainment associated with risk of exposure to tobacco advertisement in Hispanic, African American, and white individuals.^27^

At least some of the disparities across racial/ethnic groups in the burden of tobacco-related health outcomes are not associated with individuals’ choices but with higher exposure to tobacco marketing among racial/ethnic minority groups and individuals with lower socioeconomic status compared with non–racial/ethnic minority groups and individuals with higher socioeconomic status.^28,29,30^ Previous studies have shown that individuals with lower socioeconomic status and in racial/ethnic minority groups are at increased risk for exposure to point-of-sale advertising, retail displays, and coupons or discounts.^31,32^ Tobacco coupons, discounts, and advertisement are widely recognized as marketing practices that the tobacco industry uses in communities at increased risk of harm secondary to tobacco use.^33,34^ Coupons or discounts are risk factors for tobacco use^31,34,35,36,37,38^ and may be associated with tobacco disparities, given that racial/ethnic minority groups and lower socioeconomic status communities may be more likely to be targeted by tobacco marketing.^31,38,39^

In theory, at least some of the weaker than expected association of educational attainment with tobacco use among Hispanic and African American individuals may be associated with the tobacco industry’s marketing practices that disproportionately target communities of color.^31,40,41^ One study by Soneji et al^40^ suggested that the tobacco industry may specifically target Hispanic and African American individuals. The exact marketing practices that are associated with influencing people of different races, ethnicities, and educational levels are unknown, to my knowledge. I argue that differential exposure to tobacco advertisements may be associated with tobacco use by Hispanic and African American individuals, even among those with high educational attainment.^16,17^ A study by Brock et al^34^ found that as educational attainment increases, exposure to cigarette advertisement and coupons decreases. However, in the presence of weaker associations of educational attainment with exposure to tobacco advertising,^17,18,19,21,42^ highly educated Hispanic and African American individuals would likely still be exposed to tobacco advertisements at higher rates than their white peers. This may be because educational attainment has been shown to be more weakly associated with improvements in life conditions among Hispanic and African American individuals compared with non-Hispanic white individuals. In addition, African American and Hispanic individuals have been shown to remain at risk of poverty despite high educational attainment.^43^ Educational attainment has also been shown to be more strongly associated with increased income and upward social mobility among non-Hispanic white individuals than among Hispanic and African American individuals.^44,45,46^ Similarly, educational attainment has been shown to be associated with improved mental health,^23^ happiness,^47^ and impulse control^48^ among non-Hispanic white individuals compared with Hispanic and African American individuals.^45^ This study was conducted to assess if Hispanic and African American race/ethnicity are associated with higher risk of exposure to tobacco marketing regardless of level of educational attainment in a national sample of US young adults.

I expected to find an inverse association of educational attainment with exposure to tobacco advertisements and a weaker negative association of higher educational attainment with exposure to tobacco advertisements among Hispanic and African American individuals compared with non-Hispanic white individuals.

## Methods

### Design and Settings

For this cross-sectional study, I analyzed wave 1 data from the Population Assessment of Tobacco and Health (PATH) Study,^49,50^ which was conducted from 2013 to 2014. This analysis was conducted between September 20 and October 4, 2019. Jointly funded by the National Institutes of Health and the US Food and Drug Administration, the PATH Study is the primary source of epidemiological information regarding tobacco use among US residents. The PATH Study enrolled adults 18 years or older.^49,50^ This report follows the Strengthening the Reporting of Observational Studies in Epidemiology (STROBE) reporting guideline.

### Ethics

All adult participants in the PATH Study provided written informed consent. The institutional review board of Westat approved the study protocol. Data were collected, stored, and analyzed anonymously. This was a secondary analysis of fully deidentified public data, and the Charles R. Drew University of Medicine and Science exempted this study from an institutional review as it was considered non–human subject research. Westat did not have any role in this study.

### Sampling, Sample, and Analytical Sample

The PATH sample included civilian, noninstitutionalized, US adults 18 years or older. A multistage sampling design was used (ie, 4-stage probability sampling). First, a stratified sample of geographical primary sampling units were drawn. Second, smaller geographical segments in each primary sampling unit were selected. Third, residential addresses (ie, households) were selected using US Postal Service data files. Fourth, 1 individual was selected from each sampled household.^49,50^ The analysis included individuals who were aged 18 to 24 years (ie, young adults), had valid data on tobacco advertisement exposure, and were either white or African American and Hispanic or non-Hispanic. People with mixed (multiple) race/ethnicity, missing data on race/ethnicity, unknown race/ethnicity, or who identified as another race/ethnicity were excluded.

### Variables

The study variables included demographic factors (ie, race/ethnicity, sex, and region), socioeconomic status (ie, educational attainment and poverty status), and tobacco advertisement exposures. Variables were all measured at an individual level.

#### Race/Ethnicity

Race/ethnicity was self-identified. For the purpose of this study, race and ethnicity were operationalized as 2 dichotomous variables: African American vs white and Hispanic vs non-Hispanic.

#### Confounders

Sex was treated as a dichotomous variable (male, 1; female, 0). Region was defined as a categorical variable: West, South, Midwest, and Northeast. West was used as the reference group. Poverty status was defined based on the federal poverty threshold based on the household income and household size. This variable was a dichotomous variable, with 1 indicting living out of poverty and 0, living in poverty.

#### Tobacco Advertisement Exposure

The outcome was the number of different tobacco advertisements^51,52,53,54,55,56,57,58^ that were seen by the participant in the year before the survey was conducted. A total of 20 advertisements were shown to the individuals. The advertisements were selected randomly and included Marlboro, Camel, Newport, Wave, American Spirit, Winston, American Gold, Pall Mall, L & M, Grizzly, Copenhagen, Blue Cig, Green Smoke, Swisher Sweets, Ploom, Triple Crown, General Swedish Snus, Apollo, and NJOY. After each advertisement was shown, the participant was asked “In the past 12 months, have you seen this advertisement before this study?” The 2 possible responses for each item were yes or no. The total score was calculated (range, 0-20), with a higher score indicating higher exposure to tobacco advertisement. Although the measure is highly reliant on recalling, it is unlikely that the person would recall an advertisement without being exposed to it. Tobacco advertisement exposure was treated as a binary outcome and as a count variable, with a higher score indicating more exposure to tobacco advertisements.

#### Educational Attainment

For the main analysis, educational attainment was operationalized as a 3-level variable: (1) less than high school or general educational development diploma; (2) high school graduate, some college (no degree), or associate’s degree; and (3) bachelor’s degree or advanced degree. A 6-level educational level variable was used for the sensitivity analysis: (1) less than high school, (2) general educational development diploma, (3) high school graduate, (4) some college (no degree) or associate’s degree, (5) bachelor’s degree, and (6) advanced degree. Education ranged from 1 to 6 as a continuous measure, with a higher score indicating higher educational attainment.

### Statistical Analysis

Data were analyzed using SPSS statistical software version 23.0 (IBM Corp). The PATH Study data were adjusted for complex survey design, including examination of the distribution of variables and ruling out collinearity between variables, such as race/ethnicity, educational attainment, and poverty status, using Spearman correlation tests. For multivariable analysis, logistic regression models were fitted with any tobacco advertisement exposure as the outcome. For sensitivity analysis, a Poisson regression model was used, which also confirmed the observed interaction. Models in the pooled sample were performed without and with interaction terms between race/ethnicity and educational attainment (ie, African American × high school graduation, Hispanic × high school graduation, African American × college graduation, and Hispanic × college graduation). From the logistic regression model, odds ratios (ORs), SEs, 95% CIs, and *P* values were calculated. *P* values were 2-sided, and statistical significance was set at less than .05. None of the study variables had missing data.

## Results

This study included 6700 young adults (3366 [50.2%] men) between ages 18 and 24 years who provided a valid answer about their tobacco advertisement exposure during the past 12 months. Most participants were non-Hispanic (5257 participants [78.9%]) and white (5394 participants [80.5%]); 1443 participants (21.5%) were Hispanic. Educational levels included 1167 participants (17.4%) with less than a high school diploma, 4812 participants (71.8%) who were high school graduates, and 721 participants (10.8%) who were college graduates. A total of 4728 participants (70.6%) reported exposure to tobacco advertisements (Table 1).

**Table 1.** Descriptive Statistics Summary of the Overall Sample

Characteristic	Participants, No. (%)				
	All (n = 6700)	White (n = 5394)	African American (n = 1306)	Non-Hispanic (n = 5257)	Hispanic (n = 1443)
Race					
White	5394 (80.5)	5394 (100.0)	NA	4072 (77.5)	1322 (91.6)
Black	1306 (19.5)	NA	1306 (100.0)	1185 (22.5)	121 (8.4)
Ethnicity					
Non-Hispanic	5257 (78.5)	4072 (75.5)	1185 (90.7)	5257 (100.0)	NA
Hispanic	1443 (21.5)	1322 (24.5)	121 (9.3)	NA	1443 (100.0)
Sex					
Women	3334 (49.8)	2605 (48.3	729 (55.8)	2630 (50.0)	704 (48.8)
Men	3366 (50.2)	2789 (51.7)	577 (44.2)	2627 (50.0)	739 (51.2)
Region					
Northeast	959 (14.3)	777 (14.4)	182 (13.9)	813 (15.5)	146 (10.1)
Midwest	1591 (23.7)	1335 (24.7)	256 (19.6)	1466 (27.9)	125 (8.7)
South	2659 (39.7)	1909 (35.4)	750 (57.4)	2130 (40.5)	529 (36.7)
West	1491 (22.3)	1373 (25.5)	118 (9.0)	848 (16.1)	643 (44.6)
Poverty status					
In poverty	3326 (49.6)	2500 (46.3)	826 (63.2)	2469 (47.0)	857 (59.4)
Out of poverty	3374 (50.4)	2894 (53.7)	480 (36.8)	2788 (53.0)	586 (40.6)
Educational attainment					
<High school graduate	1167 (17.4)	901 (16.7)	266 (20.4)	833 (15.8)	334 (23.1)
High school graduate	4812 (71.8)	3852 (71.4)	960 (73.5)	3780 (71.9)	1032 (71.5)
College graduate	721 (10.8)	641 (11.9)	80 (6.1)	644 (12.3)	77 (5.3)
Tobacco advertisement exposure					
No	1972 (29.4)	1663 (30.8)	309 (23.7)	1551 (29.5)	421 (29.2)
Yes	4728 (70.6)	3731 (69.2)	997 (76.3)	3706 (70.5)	1022 (70.8)

Abbreviation: NA, not applicable

### Advertisement Exposure

Table 2 provides frequency and percentage of exposure to tobacco advertisements during the past 12 months by the intersection of race/ethnicity and educational attainment. In the total sample, compared with individuals without high school education, those who had high school graduation, and those who were college graduates were associated with a stepwise reduction in exposure to tobacco advertisements (76.4% vs 71.8% vs 53.1%; *P* < .001). The same associated stepwise increase in exposure to tobacco advertisements was observed in white (75.7% vs 70.5% vs 51.8%; *P* < .001) and non-Hispanic (78.3% vs 71.8% vs 52.8%; *P* < .001) individuals. Although still statistically significant, this associated stepwise reduction was weaker and showed a different pattern in African American (78.9% vs 76.7% vs 63.8%; *P* = .005) and Hispanic (71.9% vs 71.6% vs 55.8%; *P* = .02) individuals. The rates of tobacco advertisement exposure were comparable between individuals with vs without high school graduation among African American (76.7% vs 78.9%) and Hispanic (71.6% vs 71.9%) individuals (Table 2).

**Table 2.** Advertisement Exposure Based on the Intersection of Race/Ethnicity and Educational Attainment

Educational Attainment	Participants With Tobacco Advertisement Exposure, No. (%)				
	All (n = 6700)	White (n = 5394)	African American (n = 1306)	Non-Hispanic (n = 5257)	Hispanic (n = 1443)
< High school graduate	892 (76.4)	682 (75.7)	210 (78.9)	652 (78.3)	240 (71.9)
High school graduate	3453 (71.8)	2717 (70.5)	736 (76.7)	2714 (71.8)	739 (71.6)
College graduate	383 (53.1)	332 (51.8)	51 (63.8)	340 (52.8)	43 (55.8)
*P* Value^a^	<.001	<.001	.005	<.001	.02

^a^ χ^2^ for comparisons across educational levels within group of race/ethnicity.

### Logistic Regression Models

Table 3 presents the summary of the results of 2 logistic regression models with categorical educational attainment as the independent variable and any exposure to tobacco advertisement during the past 12 months as the dependent variable. Both models were performed in the overall sample. Model 1 did not have any interaction term. Model 2 included 4 interaction terms: African American race with high school graduation, Hispanic ethnicity with high school graduation, African American race with college graduation, and Hispanic ethnicity with college graduation.

**Table 3.** Logistic Regression on Tobacco Advertisement Exposure in the Pooled Sample

Variable	Model 1^a^			Model 2^b^		
	*B* (SE)	OR (95% CI)	*P* Value	*B* (SE)	OR (95% CI)	*P* Value
Hispanic ethnicity	−0.01 (0.07)	0.99 (0.86-1.14)	.92	−0.31 (0.15)	0.73 (0.54-0.99)	.04
African American race	0.31 (0.07)	1.36 (1.18-1.58)	<.001	0.11 (0.17)	1.11 (0.79-1.57)	.54
Men	0.06 (0.05)	1.06 (0.95-1.18)	.31	0.06 (0.05)	1.06 (0.95-1.18)	.30
Region			.89			.84
West	1 [Reference]	1 [Reference]		1 [Reference]	1 [Reference]	
South	−0.02 (0.09)	0.98 (0.82-1.17)	.81	−0.02 (0.09)	0.98 (0.82-1.17)	.80
Midwest	−0.05 (0.08)	0.96 (0.81-1.13)	.59	−0.05 (0.08)	0.95 (0.80-1.12)	.53
Northeast	−0.07 (0.09)	0.93 (0.78-1.12)	.46	−0.08 (0.09)	0.93 (0.77-1.11)	.42
Living out of poverty	0 (0.06)	1.00 (0.90-1.12)	.95	0 (0.06)	1.00 (0.90-1.12)	.96
Educational Attainment						
Highschool graduate	−0.24 (0.08)	0.79 (0.68-0.92)	.002	−0.39 (0.11)	0.68 (0.55-0.83)	<.001
College graduate	−0.79 (0.08)	0.46 (0.39-0.54)	<.001	−0.81 (0.09)	0.44 (0.37-0.53)	<.001
Interaction of Hispanic ethnicity with educational attainment						
Highschool graduate	NA	NA	NA	0.36 (0.17)	1.44 (1.03-2.01)	.03
College graduate	NA	NA	NA	0.12 (0.26)	1.13 (0.68-1.86)	.65
Interaction of African American race with educational attainment						
Highschool graduate	NA	NA	NA	0.22 (0.19)	1.25 (0.85-1.82)	.26
College graduate	NA	NA	NA	0.19 (0.26)	1.21 (0.72-2.01)	.47
Intercept	1.12 (0.11)	3.07	<.001	1.26 (0.13)	3.54	<.001

Abbreviations: *B*, regression coefficient; OR, odds ratio; NA, not applicable.

^a^ Examines main variables only.

^b^ Examines model 1 and interactions only.

Model 1 showed an inverse association of educational attainment with tobacco advertisement exposure for high school graduates (OR, 0.79; 95% CI, 0.68-0.92; *P* = .002) and for college graduates (OR, 0.46; 95% CI, 0.39-0.54; *P* < .001) after adjustment for all covariates. Based on model 2, Hispanic ethnicity showed a significant interaction association with high school graduation for increased exposure to tobacco advertisements (OR, 1.44; 95% CI, 1.03-2.01; *P* < .001), suggesting that the inverse association of high school graduation with tobacco advertisement exposure is significantly weaker for Hispanic than for non-Hispanic individuals (Table 3).

### Sensitivity Analysis

Table 4 presents the summary of the results of 2 Poisson regression models with continuous educational attainment as the independent variable and exposure to tobacco advertisement as the dependent variable. Both models were performed in the overall sample. Model 1 did not have any interaction terms. Model 2 also included 2 interaction terms: Hispanic ethnicity with educational attainment and African American race with educational attainment.

**Table 4.** Poisson Regression on Tobacco Advertisement Exposure in the Pooled Sample

Variable	Model 1^a^		Model 2^b^	
	*B* (SE) [95% CI]	*P* Value	*B* (SE) [95% CI]	*P* Value
Hispanic ethnicity	−0.02 (0.02) [−0.06 to 0.02]	.37	−0.20 (0.05) [−0.30 to −0.10]	<.001
African American race	0.07 (0.02) [0.03 to 0.11]	<.001	−0.24 (0.05) [−0.34 to −0.14]	<.001
Men	0.03 (0.02) [0 to 0.06]	.03	0.03 (0.02) [0.01 to 0.06]	.02
Region				
West	0.03 (0.03) [−0.02 to 0.09]	.22	0.03 (0.03) [−0.02 to 0.09]	.20
South	0.13 (0.02) [0.08 to 0.18]	<.001	0.13 (0.02) [0.08 to 0.18]	<.001
Midwest	0.10 (0.03) [0.05 to 0.15]	<.001	0.10 (0.03) [0.05 to 0.15]	<.001
Northeast				
Educational attainment	−0.15 (0.01) [−0.16 to −0.14]	<.001	−0.18 (0.01) [−0.20 to −0.17]	<.001
Living out of poverty	−0.17 (0.02) [−0.21 to −0.14]	<.001	−0.17 (0.02) [−0.20 to −0.13]	<.001
Interaction of African American race × educational attainment	NA	NA	0.10 (0.02) [0.07 to 0.13]	<.001
Interaction of Hispanic ethnicity × educational attainment	NA	NA	0.06 (0.02) [0.03 to 0.09]	<.001
Intercept	1.42 (0.03) [1.36 to 1.48]	<.001	1.53 (0.04) [1.46 to 1.60)]	<.001

Abbreviations: *B*, regression coefficient; NA, not applicable.

^a^ Examines main variables only.

^b^ Examines model 1 and interactions only.

Model 1 showed an inverse association of educational attainment with tobacco advertisement exposure (*B* = −0.17; 95% CI, −0.21 to −0.14; *P* < .001), after adjustment for all covariates. Based on model 2, there were significant interactions with educational attainment on tobacco advertisement exposure associated with African American (*B* = 0.10; 95% CI, 0.07-0.13; *P* < .001) or Hispanic (*B* = 0.06; 95% CI, 0.03-0.09; *P* < .001) race/ethnicity. This finding suggests that the inverse associations of educational attainment with tobacco advertisement exposure are weaker among Hispanic and African American individuals than among white and non-Hispanic individuals (Table 4).

## Discussion

This cross-sectional study found that high school and college graduation were associated with a stepwise reduction in exposure to tobacco advertisement in the overall sample of young adults. However, the inverse association of high school graduation with tobacco advertisement exposure was weaker among Hispanic individuals than non-Hispanic individuals. Thus, in line with my hypothesis, the weaker inverse association of educational attainment with tobacco advertisement exposure meant that Hispanic high school graduates reported higher-than-expected exposure to tobacco advertisements.

At least some of the racial/ethnic tobacco burden disparities are not associated with individuals’ choices but with higher exposure to tobacco marketing among communities of racial/ethnic minority groups and communities with lower socioeconomic status compared with communities of non–racial/ethnic minority groups and communities with higher socioeconomic status.^28,29,30^ People of color and individuals who live in lower socioeconomic status areas are at an increased risk for exposure to point-of-sale advertising, retail displays, and coupons or discounts.^31,32^ Tobacco coupons, discounts, and advertisements specifically target these communities.^33,34^ Advertisements and coupons or discounts are among the main risk factors of tobacco use^31,34,35,36,37,38^ and are potential contributors to tobacco use disparities.^31,38,39^

Marketing practices may be associated with the higher tobacco risk associated with lower socioeconomic status and with African American and Hispanic race/ethnicity. One study in 6 neighborhoods in Boston, Massachusetts,^59^ found that people were heavily exposed to outdoor cigarette advertising, particularly people in areas with significant African American and Hispanic or Latino populations and with lower socioeconomic status. The results of this study provide some suggestive evidence for high exposure to tobacco advertisements in Hispanic and African American populations, regardless of socioeconomic status. Other studies have also suggested that the tobacco industry disproportionately targets these communities,^14,53,60,61,62,63,64,65,66^ which may increase the risk of tobacco use in their residents. Such place-based activity may also impose risk to high socioeconomic status African American and Hispanic individuals who live in communities that include predominantly African American and Hispanic populations.

Previous research has shown disproportionately high risk of tobacco use in highly educated and high–socioeconomic status Hispanic and African American individuals, across tobacco products.^16,17,21,67^ Highly educated Hispanic and African American individuals are also exposed to higher levels of secondhand tobacco smoke inside their homes^68^ and at work^26^ compared with white individuals. Thus, highly educated racial/ethnic minority groups remain at high risk of chronic medical conditions, such as chronic obstructive pulmonary disease,^69^ asthma,^70^ and hypertension.^22^ Similarly, rates of hospitalization^71^ and mortality^72^ are higher in highly educated racial/ethnic minority groups than in racial/ethnic majority groups. This pattern of weaker-than-expected associations of educational attainment in the lives of people of color compared with their white peers has been described previously as “minorities’ diminished returns.”^18,20^ The findings of this study suggest in a similar pattern in that highly educated racial/ethnic minority groups had higher-than-expected rates of exposure to tobacco advertisements, disproportionate to their education level.

Weaker-than-expected associations of health outcomes with socioeconomic status indicators are not limited to tobacco outcomes and have been documented for diet,^73^ exercise,^74^ obesity,^42,75^ depression,^76^ anxiety,^77^ self-rated health,^19,24^ and health care use,^78^ and have been described in children,^70^ youths,^79,80^ adults,^20^ and older adults.^81^ Similar patterns are also shown for marginalizing social identities other than race/ethnicity,^17,19,42,71,82,83^ such as sexual orientation.^84,85,86^ The robust and systemic nature of the weaker than expected associations of health outcomes with education suggests that socioeconomic status may lose some of its protective associations among people marginalized by society, regardless of socioeconomic status, marginalizing identity, and outcome.

Given the existing weaker-than-expected associations of health outcomes with education for racial/ethnic minority groups, racial/ethnic gaps in tobacco exposure may increase, rather than decrease, as socioeconomic status increases.^26^ Rather than socioeconomic status per se, the major risk factors for tobacco disparities in middle-class racial/ethnic minority groups^16,17^ may be residual environmental exposures that continue regardless of socioeconomic status. The findings in this study suggest that at least some of the additional risk of tobacco use in highly educated racial/ethnic minority groups may be associated with environmental risk factors for tobacco use. Increased exposure to tobacco advertisement is a form of structural and place-based discrimination that is associated with worse health and well-being of people of color in the United States.^39,41,59^ Given existing residential segregation, people of color are more likely to live in proximity to tobacco retail stores, liquor shops, and tobacco outlets.^87^ Future research is needed on societal mechanisms that expose highly educated individuals from racial/ethnic minority groups to tobacco advertisement.

If marketing is responsible for greater than expected tobacco use among highly educated racial/ethnic minority groups, then restricting marketing may be a solution to the tobacco burden disparities^14,60,61,62,63,64,65^ in racial/ethnic minority groups across socioeconomic levels, and more restrictive regulatory policies may be needed. However, even tobacco regulatory policies may be differently associated with outcomes across social groups.

### Implications

These results have some policy and public health implications. The results may encourage the Food and Drug Administration and local authorities to consider more restrictive regulation of tobacco marketing that has traditionally targeted people of color.^30,41,65,66^ Tighter restrictions on tobacco marketing in communities of color may reduce some of the tobacco-related disparities that affect high socioeconomic status racial/ethnic minority groups. Reducing disparities is a strategic priority for the Food and Drug Administration and the National Institutes of Health.^88^ Further research is needed to identify interventions, including restrictive national and local regulations, such as banning advertisements that target racial/ethnic minority groups, and to implement evidence-backed policies. A 2019 study by Feliu et al^89^ suggested that US residents favor restrictive tobacco regulations and do not consider such restrictions to be an imposition on their autonomy.

### Future Research

Future studies should specifically explore how different types of advertisements affect populations based on the intersection of race/ethnicity and socioeconomic status. Researchers may also study differential associations of tobacco policies, such as banning certain marketing practices, point-of-sale advertisements, flavoring, and direct mailing, with tobacco outcomes across diverse racial/ethnic groups. As this study only focused on the role of educational attainment as a marker of socioeconomic status, future research should also explore associations of health outcomes with wealth, income, employment, occupational prestige, marital status, and area-level socioeconomic status. Additionally, this study only included African American, Hispanic, non-Hispanic, and white individuals. Future investigations may explore other minority groups, such as other racial/ethnic groups and immigrants. Future research may go beyond dichotomous outcomes and capture the frequency of exposure to advertisement.

This study was performed in the general population that included both tobacco users and nonusers. Although smoking status could alter the association of education with exposure to tobacco advertisement, this study did not perform models based on smoking status for statistical power concerns, given that very few individuals were highly educated, smokers, and black or Hispanic. Future research should use larger samples to test variation of exposure to tobacco advertisements among smoking and nonsmoking adults. Additionally, this study could not explore the association based on smoking status, as the estimates were unstable and CIs were wide. Still, future research may use larger samples to explore how these processes differ for smokers and nonsmokers.

### Limitations

This study has some limitations. Given the cross-sectional design of this study, causation cannot be shown. The unbalanced sample size by ethnicity could generate differential statistical power across racial/ethnic groups. Thus, models were not performed within racial/ethnic groups. Instead, models with interaction terms in the pooled sample were performed, which is not affected by the unbalanced sample size distribution across racial/ethnic minority groups. This study did not measure all confounders, such as physical and mental health, tobacco use, and access to tobacco cessation programs. The PATH Study also did not have data on details of exposure to tobacco advertisement. These factors may explain why and how highly educated racial/ethnic minority groups may remain at high risk of exposure to tobacco marketing. Additionally, similar to any other study relying on self-reported measures, this study is prone to measurement bias. This problem is not limited to advertisement exposure, and any retrospective measurement would be accompanied by some degree of recall bias. Despite this limitation, many scholars have measured exposure by relying on self-report and recall as exposure to tobacco advertisement.^51,52,53,54,55,56,57,58^

This reliance on self-report may become a problem if social groups differ in how they recall advertisements that they are exposed to. However, I am not aware of any previous study showing cross–racial/ethnic variation in the validity of this measure. Recognizing advertisements may not function as an exposure to marketing; however, prior research has used advertisement recall as a measure of exposure to advertisement.^51,52,53,54,55,56,57,58^ Interpretation of the results on recalls requires caution because the nature of the data are cross-sectional rather than longitudinal. This study could not rule out the likelihood of cultural or behavioral differences that may have contributed to differential recall despite equal exposure to advertisements. Additionally, this study did not measure frequency of exposure to tobacco advertisement but rather number of types of advertisements. If someone saw a particular advertisement every day for 12 months, this would be counted as yes with a score of 1, and if a person saw 3 different types of advertisements over the course of 12 months, the score would be 3. As a nationally representative sample was used, these results are likely generalizable to US young adults, but there is still a need to study geographic variation of exposure to tobacco advertisements by race/ethnicity and socioeconomic status.

## Conclusions

In the United States, racial/ethnic minority status is associated with weakened inverse association of educational attainment with exposure to tobacco advertisement. While highly educated people are less likely to be exposed to tobacco advertisements, this pattern is less true for highly educated Hispanic and African American individuals than non-Hispanic white individuals. Additional exposure to tobacco advertisements may, in part, explain the results of previous studies on higher-than-expected tobacco risk among highly educated racial/ethnic minority groups.

## References

[1] Ellickson PL, Orlando M, Tucker JS, Klein DJ From adolescence to young adulthood: racial/ethnic disparities in smoking. Am J Public Health. 2004;94(2):-. doi:10.2105/AJPH.94.2.293 14759945 PMC1448246

[2] Centers for Disease Control and Prevention Racial disparities in smoking-attributable mortality and years of potential life lost—Missouri, 2003-2007. MMWR Morb Mortal Wkly Rep. 2010;59(46):1518-1522. 21102406

[3] Trinidad DR, Pérez-Stable EJ, White MM, Emery SL, Messer K A nationwide analysis of US racial/ethnic disparities in smoking behaviors, smoking cessation, and cessation-related factors. Am J Public Health. 2011;101(4):699-706. doi:10.2105/AJPH.2010.191668 21330593 PMC3052356

[4] Soulakova JN, Huang H, Crockett LJ Racial/ethnic disparities in consistent reporting of smoking-related behaviors. J Addict Behav Ther Rehabil. 2015;4(4). doi:10.4172/2324-9005.1000147 27088100 PMC4831627

[5] Blumenthal DS Racial and ethnic disparities in smoking prevalence in Israel and the United States: progress to date and prospects for the future. Isr J Health Policy Res. 2017;6(1):51. doi:10.1186/s13584-017-0177-9 28969689 PMC5625817

[6] Cokkinides VE, Halpern MT, Barbeau EM, Ward E, Thun MJ Racial and ethnic disparities in smoking-cessation interventions: analysis of the 2005 National Health Interview Survey. Am J Prev Med. 2008;34(5):404-412. doi:10.1016/j.amepre.2008.02.003 18407007

[7] Tran ST, Rosenberg KD, Carlson NE Racial/ethnic disparities in the receipt of smoking cessation interventions during prenatal care. Matern Child Health J. 2010;14(6):901-909. doi:10.1007/s10995-009-0522-x 19795200

[8] Greaves L, Hemsing N Women and tobacco control policies: social-structural and psychosocial contributions to vulnerability to tobacco use and exposure. Drug Alcohol Depend. 2009;104(suppl 1):S121-S130. doi:10.1016/j.drugalcdep.2009.05.001 19520523

[9] Wallace JM Jr, Vaughn MG, Bachman JG, O’Malley PM, Johnston LD, Schulenberg JE Race/ethnicity, socioeconomic factors, and smoking among early adolescent girls in the United States. Drug Alcohol Depend. 2009;104(suppl 1):S42-S49. doi:10.1016/j.drugalcdep.2009.06.007 19628345 PMC2732752

[10] Laveist TA, Thorpe RJ Jr, Mance GA, Jackson J Overcoming confounding of race with socio-economic status and segregation to explore race disparities in smoking. Addiction. 2007;102(suppl 2):65-70. doi:10.1111/j.1360-0443.2007.01956.x 17850615

[11] Reid JL, Hammond D, Driezen P Socio-economic status and smoking in Canada, 1999-2006: has there been any progress on disparities in tobacco use? Can J Public Health. 2010;101(1):73-78. doi:10.1007/BF03405567 20364543 PMC6973977

[12] Zhang X, Martinez-Donate AP, Jones NR Educational disparities in home smoking bans among households with underage children in the United States: can tobacco control policies help to narrow the gap? Nicotine Tob Res. 2013;15(12):1978-1987. doi:10.1093/ntr/ntt090 23902734

[13] Reimer RA, Gerrard M, Gibbons FX Racial disparities in smoking knowledge among current smokers: data from the health information national trends surveys. Psychol Health. 2010;25(8):943-959. doi:10.1080/08870440902935913 20204962

[14] Rock VJ, Davis SP, Thorne SL, Asman KJ, Caraballo RS Menthol cigarette use among racial and ethnic groups in the United States, 2004-2008. Nicotine Tob Res. 2010;12(suppl 2):S117-S124. doi:10.1093/ntr/ntq204 21177368

[15] Drope J, Liber AC, Cahn Z, Who’s still smoking: disparities in adult cigarette smoking prevalence in the United States. CA Cancer J Clin. 2018;68(2):106-115. doi:10.3322/caac.21444 29384589

[16] Assari S, Mistry R Educational attainment and smoking status in a national sample of American adults: evidence for the blacks’ diminished return. Int J Environ Res Public Health. 2018;15(4):E763. doi:10.3390/ijerph15040763 29659482 PMC5923805

[17] Assari S, Mistry R Diminished return of employment on ever smoking among Hispanic whites in Los Angeles. Health Equity. 2019;3(1):138-144. doi:10.1089/heq.2018.0070 31289772 PMC6608689

[18] Assari S Health disparities due to diminished return among black Americans: public policy solutions. Soc Issues Policy Rev. 2018;12(1):112-145. doi:10.1111/sipr.12042

[19] Assari S Blacks’ diminished return of education attainment on subjective health: mediating effect of income. Brain Sci. 2018;8(9):E176. doi:10.3390/brainsci8090176 30213135 PMC6162786

[20] Assari S Unequal gain of equal resources across racial groups. Int J Health Policy Manag. 2018;7(1):1-9. doi:10.15171/ijhpm.2017.90 29325397 PMC5745862

[21] Assari S, Farokhnia M, Mistry R Education attainment and alcohol binge drinking: diminished returns of Hispanics in Los Angeles. Behav Sci (Basel). 2019;9(1):E9. doi:10.3390/bs9010009 30646592 PMC6359422

[22] Assari S Socioeconomic determinants of systolic blood pressure: minorities’ diminished returns. J Health Econ Dev. 2019;1(1):1-11. 31428747

[23] Assari S Parental educational attainment and mental well-being of college students: diminished returns of blacks. Brain Sci. 2018;8(11):E193. doi:10.3390/brainsci8110193 30380617 PMC6266217

[24] Assari S, Lapeyrouse LM, Neighbors HW Income and self-rated mental health: diminished returns for high income black Americans. Behav Sci (Basel). 2018;8(5):E50. doi:10.3390/bs8050050 29772799 PMC5981244

[25] Ruglass LM, Root JC, Dambreville N, Smoking policies in the home have less influence on cigarettes per day and nicotine dependence level among African American than white smokers: a cross-sectional analysis. J Natl Med Assoc. 2019;S0027-9684(19)30091-4. doi:10.1016/j.jnma.2019.07.002 31375277 PMC6925645

[26] Assari S, Bazargan M Unequal effects of educational attainment on workplace exposure to second-hand smoke by race and ethnicity: minorities’ diminished returns in the National Health Interview Survey (NHIS). J Med Res Innov. 2019;3(2):e000179. doi:10.32892/jmri.179 31404444 PMC6688774

[27] Simon P, Camenga DR, Morean ME, Socioeconomic status and adolescent e-cigarette use: the mediating role of e-cigarette advertisement exposure. Prev Med. 2018;112:193-198. doi:10.1016/j.ypmed.2018.04.019 29673887 PMC6007030

[28] Terry-McElrath YM, Wakefield MA, Emery S, State anti-tobacco advertising and smoking outcomes by gender and race/ethnicity. Ethn Health. 2007;12(4):339-362. doi:10.1080/13557850701300723 17701761

[29] Keeler C, Max W, Yerger V, Yao T, Ong MK, Sung HY The association of menthol cigarette use with quit attempts, successful cessation, and intention to quit across racial/ethnic groups in the United States. Nicotine Tob Res. 2017;19(12):1450-1464. 27613927 10.1093/ntr/ntw215 PMC6251684

[30] Giovenco DP, Spillane TE, Merizier JM Neighborhood differences in alternative tobacco product availability and advertising in New York City: implications for health disparities. Nicotine Tob Res. 2019;21(7):896-902. doi:10.1093/ntr/nty244 30452712 PMC6588385

[31] Lewis MJ, Delnevo CD, Slade J Tobacco industry direct mail marketing and participation by New Jersey adults. Am J Public Health. 2004;94(2):257-259. doi:10.2105/AJPH.94.2.257 14759937 PMC1448238

[32] Audrain-McGovern J, Tercyak KP, Shields AE, Bush A, Espinel CF, Lerman C Which adolescents are most receptive to tobacco industry marketing: implications for counter-advertising campaigns. Health Commun. 2003;15(4):499-513. doi:10.1207/S15327027HC1504_07 14527869

[33] Anderson SJ Marketing of menthol cigarettes and consumer perceptions: a review of tobacco industry documents. Tob Control. 2011;20(suppl 2):ii20-ii28. doi:10.1136/tc.2010.041939 21504928 PMC3088454

[34] Brock B, Schillo BA, Moilanen M Tobacco industry marketing: an analysis of direct mail coupons and giveaways. Tob Control. 2015;24(5):505-508. doi:10.1136/tobaccocontrol-2014-051602 25052861

[35] Choi K, Soneji S, Tan ASL Receipt of tobacco direct mail coupons and changes in smoking status in a nationally representative sample of US adults. Nicotine Tob Res. 2018;20(9):1095-1100. doi:10.1093/ntr/ntx141 30124987 PMC6093365

[36] Brock B, Carlson SC, Moilanen M, Schillo BA Reaching consumers: how the tobacco industry uses email marketing. Prev Med Rep. 2016;4:103-106. doi:10.1016/j.pmedr.2016.05.020 27413669 PMC4929071

[37] Huang J, Tauras J, Chaloupka FJ The impact of price and tobacco control policies on the demand for electronic nicotine delivery systems. Tob Control. 2014;23(suppl 3):iii41-iii47. doi:10.1136/tobaccocontrol-2013-051515 24935898 PMC4145658

[38] Choi K, Forster JL Frequency and characteristics associated with exposure to tobacco direct mail marketing and its prospective effect on smoking behaviors among young adults from the US Midwest. Am J Public Health. 2014;104(11):2179-2183. doi:10.2105/AJPH.2014.302123 25211739 PMC4192087

[39] Brown-Johnson CG, England LJ, Glantz SA, Ling PM Tobacco industry marketing to low socioeconomic status women in the USA. Tob Control. 2014;23(e2):e139-e146. doi:10.1136/tobaccocontrol-2013-051224 24449249 PMC4105326

[40] Soneji S, Knutzen KE, Tan ASL, Online tobacco marketing among US adolescent sexual, gender, racial, and ethnic minorities. Addict Behav. 2019;95:189-196. doi:10.1016/j.addbeh.2019.03.015 30954888 PMC6545129

[41] Moore DJ, Williams JD, Qualls WJ Target marketing of tobacco and alcohol-related products to ethnic minority groups in the United States. Ethn Dis. 1996;6(1-2):83-98. 8882838

[42] Assari S, Thomas A, Caldwell CH, Mincy RB Blacks’ diminished health return of family structure and socioeconomic status: 15 years of follow-up of a national urban sample of youth. J Urban Health. 2018;95(1):21-35. doi:10.1007/s11524-017-0217-3 29230628 PMC5862702

[43] Assari S Parental education better helps white than black families escape poverty: National Survey of Children’s Health. Economies. 2018;6(2):30. doi:10.3390/economies6020030 PMC6023379 29895767

[44] Assari S, Preiser B, Kelly M Education and income predict future emotional well-being of whites but not blacks: a ten-year cohort. Brain Sci. 2018;8(7):E122. doi:10.3390/brainsci8070122 29966278 PMC6070982

[45] Assari S Parental education attainment and educational upward mobility: role of race and gender. Behav Sci (Basel). 2018;8(11):107. doi:10.3390/bs8110107 30469353 PMC6262323

[46] Assari S Parental educational attainment and academic performance of American college students: blacks’ diminished returns. J Health Econ Dev. 2019;1(1):21-31. 31372601 PMC6673665

[47] Assari S Race, education attainment, and happiness in the United States. Int J Epidemiol Res. 2019;6(2):76-82. doi:10.15171/ijer.2019.14 31363495 PMC6666429

[48] Assari S, Caldwell CH, Mincy R Family socioeconomic status at birth and youth impulsivity at age 15: blacks’ diminished return. Children (Basel). 2018;5(5):E58. doi:10.3390/children5050058 29724004 PMC5977040

[49] Tourangeau R, Yan T, Sun H, Hyland A, Stanton CA Population Assessment of Tobacco and Health (PATH) reliability and validity study: selected reliability and validity estimates. Tob Control. 2019;28(6):663-668. doi:10.1136/tobaccocontrol-2018-054561 30297373

[50] Hyland A, Ambrose BK, Conway KP, Design and methods of the Population Assessment of Tobacco and Health (PATH) Study. Tob Control. 2017;26(4):371-378. doi:10.1136/tobaccocontrol-2016-052934 27507901 PMC5299069

[51] Duke JC, Lee YO, Kim AE, Exposure to electronic cigarette television advertisements among youth and young adults. Pediatrics. 2014;134(1):e29-e36. doi:10.1542/peds.2014-0269 24918224

[52] Pierce JP, Sargent JD, Portnoy DB, Association between receptivity to tobacco advertising and progression to tobacco use in youth and young adults in the PATH Study. JAMA Pediatr. 2018;172(5):444-451. doi:10.1001/jamapediatrics.2017.5756 29582078 PMC5875336

[53] Moran MB, Heley K, Pierce JP, Niaura R, Strong D, Abrams D Ethnic and socioeconomic disparities in recalled exposure to and self-reported impact of tobacco marketing and promotions. Health Commun. 2019;34(3):280-289. doi:10.1080/10410236.2017.1407227 29236530 PMC6004334

[54] Nicksic NE, Snell LM, Barnes AJ Does exposure and receptivity to e-cigarette advertisements relate to e-cigarette and conventional cigarette use behaviors among youth: results from wave 1 of the Population Assessment of Tobacco and Health Study. J Appl Res Child. 2017;8(2):3 https://eric.ed.gov/?id=EJ1188554. Accessed November 27, 2019.

[55] Agaku IT, Ayo-Yusuf OA The effect of exposure to pro-tobacco advertising on experimentation with emerging tobacco products among US adolescents. Health Educ Behav. 2014;41(3):275-280. doi:10.1177/1090198113511817 24347143

[56] Stevens P, Carlson LM, Hinman JM An analysis of tobacco industry marketing to lesbian, gay, bisexual, and transgender (LGBT) populations: strategies for mainstream tobacco control and prevention. Health Promot Pract. 2004;5(3)(suppl):129S-134S. doi:10.1177/1524839904264617 15231106

[57] Dilley JA, Spigner C, Boysun MJ, Dent CW, Pizacani BA Does tobacco industry marketing excessively impact lesbian, gay and bisexual communities? Tob Control. 2008;17(6):385-390. doi:10.1136/tc.2007.024216 18723561

[58] Pierce JP, Sargent JD, White MM, Receptivity to tobacco advertising and susceptibility to tobacco products. Pediatrics. 2017;139(6):e20163353. doi:10.1542/peds.2016-3353 28562266 PMC5470502

[59] Pucci LG, Joseph HM Jr, Siegel M Outdoor tobacco advertising in six Boston neighborhoods: evaluating youth exposure. Am J Prev Med. 1998;15(2):155-159. doi:10.1016/S0749-3797(98)00034-8 9713672

[60] Soneji SS, Knutzen KE, Villanti AC Use of flavored e-cigarettes among adolescents, young adults, and older adults: findings from the Population Assessment for Tobacco and Health Study. Public Health Rep. 2019;134(3):282-292. doi:10.1177/0033354919830967 30857471 PMC6505324

[61] Schneller LM, Bansal-Travers M, Goniewicz ML, McIntosh S, Ossip D, O’Connor RJ Use of flavored electronic cigarette refill liquids among adults and youth in the US: results from wave 2 of the Population Assessment of Tobacco and Health Study (2014-2015). PLoS One. 2018;13(8):e0202744. doi:10.1371/journal.pone.0202744 30138412 PMC6107209

[62] Soulakova JN, Danczak RR Impact of menthol smoking on nicotine dependence for diverse racial/ethnic groups of daily smokers. Healthcare (Basel). 2017;5(1):E2. doi:10.3390/healthcare5010002 28085040 PMC5371908

[63] Levy DT, Mays D, Boyle RG, Tam J, Chaloupka FJ The effect of tobacco control policies on US smokeless tobacco use: a structured review. Nicotine Tob Res. 2017;20(1):3-11. doi:10.1093/ntr/ntw291 27798090 PMC5896466

[64] Jones MR, Apelberg BJ, Tellez-Plaza M, Samet JM, Navas-Acien A Menthol cigarettes, race/ethnicity, and biomarkers of tobacco use in US adults: the 1999-2010 National Health and Nutrition Examination Survey (NHANES). Cancer Epidemiol Biomarkers Prev. 2013;22(2):224-232. doi:10.1158/1055-9965.EPI-12-0912 23250935 PMC3565051

[65] Mustonen TK, Spencer SM, Hoskinson RA, Sachs DP, Garvey AJ The influence of gender, race, and menthol content on tobacco exposure measures. Nicotine Tob Res. 2005;7(4):581-590. doi:10.1080/14622200500185199 16085529

[66] Seidenberg AB, Caughey RW, Rees VW, Connolly GN Storefront cigarette advertising differs by community demographic profile. Am J Health Promot. 2010;24(6):e26-e31. doi:10.4278/ajhp.090618-QUAN-196 20594091 PMC3086453

[67] Assari S, Mistry R, Bazargan M Race, educational attainment, and e-cigarette use. Journal of Medical Research and Innovation. 2020;4(1):e000185. In press. doi:10.32892/jmri.185 PMC7034862 32090188

[68] Assari S, Bazargan M Second-hand smoke exposure at home in the United States: minorities’ diminished returns. Int J Travel Med Glob Health. In press. http://www.ijtmgh.com/article_95971.html. Posted November 5, 2019. Accessed November 27, 2019. 10.15171/IJTMGH.2019.28 32195278

[69] Assari S, Chalian H, Bazargan M High education level protects European Americans but not African Americans against chronic obstructive pulmonary disease: National Health Interview Survey. Int J Biomed Eng Clin Sci. 2019;5(2):16-23. doi:10.11648/j.ijbecs.20190502.12 31633081 PMC6800655

[70] Assari S, Moghani Lankarani M Poverty status and childhood asthma in white and black families: National Survey of Children’s Health. Healthcare (Basel). 2018;6(2):E62. doi:10.3390/healthcare6020062 29895767 PMC6023379

[71] Assari S, Bazargan M Minorities’ diminished returns of educational attainment on hospitalization risk: national Health Interview Survey (NHIS). Hosp Pract Res. 2019;4(3):86-91. doi:10.15171/hpr.2019.17 31650101 PMC6812545

[72] Assari S, Lankarani MM Race and urbanity alter the protective effect of education but not income on mortality. Front Public Health. 2016;4:100. doi:10.3389/fpubh.2016.00100 27242992 PMC4873510

[73] Assari S, Lankarani M Educational attainment promotes fruit and vegetable intake for whites but not blacks. J. 2018;1(1):29-41. doi:10.3390/j1010005 PMC6914217 31844842

[74] Assari S Educational attainment and exercise frequency in american women: blacks’ diminished returns. Womens Health Bull. 2019;6(3):e87413. doi:10.5812/whb.87413 31552286 PMC6757331

[75] Assari S Family income reduces risk of obesity for white but not black children. Children (Basel). 2018;5(6):E73. doi:10.3390/children5060073 29890778 PMC6025246

[76] Assari S High income protects whites but not African Americans against risk of depression. Healthcare (Basel). 2018;6(2):E37. doi:10.3390/healthcare6020037 29690595 PMC6023547

[77] Assari S, Caldwell CH, Zimmerman MA Family structure and subsequent anxiety symptoms; minorities’ diminished return. Brain Sci. 2018;8(6):E97. doi:10.3390/brainsci8060097 29857488 PMC6025006

[78] Assari S, Bazargan M Educational attainment better increases the chance of clinical breast exam for non-Hispanic than Hispanic American women. Hosp Pract Res. In press. http://www.jhpr.ir/article_96044.html. Posted November 9, 2019. Accessed December 4, 2019. 10.15171/HPR.2019.25 32190811

[79] Assari S, Boyce S, Bazargan M, Mincy R, Caldwell CH Unequal protective effects of parental educational attainment on the body mass index of black and white youth. Int J Environ Res Public Health. 2019;16(19):3641. doi:10.3390/ijerph16193641 31569829 PMC6801712

[80] Assari S, Caldwell CH Family income at birth and risk of attention deficit hyperactivity disorder at age 15: racial differences. Children (Basel). 2019;6(1):E10. doi:10.3390/children6010010 30646634 PMC6352113

[81] Assari S, Bazargan M Educational attainment and self-rated oral health among American older adults: Hispanics’ diminished returns. Dent J (Basel). 2019;7(4):97. doi:10.3390/dj7040097 31581515 PMC6960509

[82] Assari S Socioeconomic status and self-rated oral health: diminished return among Hispanic whites. Dent J (Basel). 2018;6(2):E11. doi:10.3390/dj6020011 29695074 PMC6023433

[83] Assari S The benefits of higher income in protecting against chronic medical conditions are smaller for African Americans than whites. Healthcare (Basel). 2018;6(1):E2. doi:10.3390/healthcare6010002 29315227 PMC5872209

[84] Assari S, Schatten HT, Arias SA, Miller IW, Camargo CA, Boudreaux ED Higher educational attainment is associated with lower risk of a future suicide attempt among non-Hispanic whites but not non-Hispanic blacks. J Racial Ethn Health Disparities. 2019;6(5):1001-1010. doi:10.1007/s40615-019-00601-z 31278625 PMC6739140

[85] Assari S, Bazargan M Educational attainment and subjective health and well-being: diminished returns of lesbian, gay, and bisexual individuals. Behav Sci (Basel). 2019;9(9):90. doi:10.3390/bs9090090 31443340 PMC6770696

[86] Assari S Education attainment and obesity: differential returns based on sexual orientation. Behav Sci (Basel). 2019;9(2):E16. doi:10.3390/bs9020016 30699932 PMC6406256

[87] Berke EM, Tanski SE, Demidenko E, Alford-Teaster J, Shi X, Sargent JD Alcohol retail density and demographic predictors of health disparities: a geographic analysis. Am J Public Health. 2010;100(10):1967-1971. doi:10.2105/AJPH.2009.170464 20724696 PMC2936987

[88] Food and Drug Administration Research priorities. https://www.fda.gov/tobacco-products/research/research-priorities. Accessed February 5, 2019.

[89] Feliu A, Filippidis FT, Joossens L, Impact of tobacco control policies on smoking prevalence and quit ratios in 27 European Union countries from 2006 to 2014. Tob Control. 2019;28(1):101-109. doi:10.1136/tobaccocontrol-2017-054119 29472445 PMC6317447

